# Supplementary material for: Limited Sensitivity of Circulating Tumor DNA Detection by Droplet Digital PCR in Non-Metastatic Operable Gastric Cancer Patients
Source: Cancers (Basel). 2019 Mar 21;11(3):396. doi: 10.3390/cancers11030396 (PMC6468548; doi:10.3390/cancers11030396)
Supplement: Supplementary file 1 [file cancers-11-00396-s001.pdf]

Supplementary Materials: Limited sensitivity of circulating tumor DNA detection by droplet digital PCR in non-metastatic operable gastric cancer patients

Luc Cabel, Charles Decraene, Ivan Bieche, Jean-Yves Pierga, Mostefa Bennamoun, David Fuks, Jean-Marc Ferraz, Marine Lefevre, Sylvain Baulande, Virginie Bernard, Sophie Vacher, Pascale Mariani, Charlotte Proudhon, Francois-Clement Bidard and Christophe Louvet

Table S1. Targeted NGS panel genes.

**Targeted NGS Panel Genes 39 Genes**		
AKT1	***HRAS***	*NOTCH2*
ALK	*KDR*	*NOTCH4*
BRAF	*KEAP1*	*NRAS*
BRCA1	*KIT*	*PDGFRA*
BRCA2	*KRAS*	*PIK3CA*
CTNNB1	*MAP2K1*	*PTEN*
EGFR	*MAP2K4*	*PTPN11*
ERBB2	*MAP3K1*	*RET*
ERBB3	*MET*	*STK11*
FBXW7	*MTOR*	*TP53*
FGFR1	*NF1*	*TSC1*
FGFR2	*NFE2L2*	*TSC2*
FGFR3	*NOTCH1*	*VHL*
